# Nitric Oxide Crosstalk With Phytohormone Is Involved in Enhancing Photosynthesis of *Tetrastigma hemsleyanum* for Photovoltaic Adaptation

**DOI:** 10.3389/fpls.2022.852956

**Published:** 2022-03-09

**Authors:** Zhuomi Xie, Chuyun Yang, Mingjie Li, Zhongyi Zhang, Yao Wu, Li Gu, Xin Peng

**Affiliations:** ^1^College of Agriculture, Fujian Agriculture and Forestry University, Fuzhou, China; ^2^Key Laboratory of Ministry of Education for Genetics, Breeding and Multiple Utilization of Crops, Fujian Agriculture and Forestry University, Fuzhou, China; ^3^College of Food Science, Ningbo Research Institute of Zhejiang University, Ningbo, China; ^4^Medicinal Plant Resource Center, Ningbo Research Institute of Traditional Chinese Medicine, Ningbo, China

**Keywords:** photovoltaic adaptation, *Tetrastigma hemsleyanum*, nitric oxide, salicylic acid, crosstalk

## Abstract

Photovoltaic agriculture is a newly emerging ecological planting pattern. In view of the adverse effect on production, a better understanding of photovoltaic adaptation responses is essential for the development of the innovative agriculture mode in sustainable crop production. Here, we investigated the impact of photovoltaic condition on endogenous hormone composition and transcriptome profile of *Tetrastigma hemsleyanum*. A total of 16 differentially accumulated phytohormones and 12,615 differentially expressed genes (DEGs) were identified. Photovoltaic adaptation significantly decreased the contents of phytohormones especially salicylic acid (SA) and jasmonic acid (JA). DEGs were the most relevant to photosynthesis and mitogen-activated protein kinase (MAPK) signaling pathway especially the key genes encoding proteins involved in photosystem I (PS I) and photosystem II (PS II) reaction center. Nitric oxide (NO), JA, and SA treatment alone significantly enhanced the photosynthetic efficiency which was decreased by exposure to photovoltaic condition, but the combined treatment of “NO + SA” could weaken the enhancement effect by regulating the expression level of *psaL, CHIL, petF1, psbQ*, and *psaE* genes. Exogenous phytohormones and NO treatment mitigated the accumulation of reactive oxygen species (ROS) and potentiated antioxidant capacity, which would be weakened by the combined treatment of “NO + SA.” SA and JA significantly decreased endogenous NO burst triggered by photovoltaic adaptation. SA might be a potent scavenger of NO and counter the restoration effect of NO on growth and photosynthetic potential in *T. hemsleyanum*. The results could provide reference for the application of phytohormones/other signaling molecules in photovoltaic agriculture.

## Introduction

A sustainable and effective supply of crops to satisfy the demand in food supply is a global challenge especially under the background of climatic variation. In recent years, as the demand for clean energy continues to grow, the photovoltaic industry has developed rapidly. To rationally strengthen the intensified utilization of land resources, a new pattern of combining photovoltaic power generation and agricultural production, namely, photovoltaic agriculture, have largely emerged. Photovoltaic materials installed on the roof of greenhouses offer a protected opportunity for crop cultivation. As the principal energy source of photosynthesis, light plays a crucial role in crop growth and development. Light quality and quantity transmitted are significantly modified by cover materials, and then, generate a series of microclimate changes. So far, photovoltaic application system research is still in the initial stage. Recent research has been mainly focused on developing innovative cover materials to improve solar energy capture and light transmission. However, limited information was available about the ecological effect of the complex planting pattern on the physiological activity of crops. Some research showed that transmitted light changed by photovoltaic materials can have negative impacts on the plant production. For instance, 50% photovoltaic materials covering the roof of the greenhouse decreased tomato production significantly (Cossu et al., 2014). In contrast, a UV transmission film promotes cucumber yield (Allardyce et al., 2017), and the light cascade technology increases the fresh weight and yield of fruit (Lemarié et al., 2018). It was presumed that the photovoltaic agriculture was probably not suitable for those crops requiring high-light intensity, however, it could be developed for the cultivation of other industrial crops. Therefore, a deeper understanding of photovoltaic materials-mediated modulation/regulation of gene expression, protein, and metabolite composition in plants is essential for the development of the innovative complex ecological agriculture mode in sustainable crop production.

The metabolic processes of plants would become largely affected when subjected to abiotic stress. These adaptive responses are mediated through inducing a series of different cellular and physiological changes. Some reactive chemical species, such as reactive oxygen species (ROS), reactive nitrogen species (RNS), are generated at low concentrations and act as signaling molecules regulating secondary messengers (Zhou et al., 2021a), and interacts with other signaling components (Farnese et al., 2016; Singhal et al., 2021). There is quite a number of research indicating that ROS in plant cells are involved in various aspects of physiological responses (Campos et al., 2019; Jahan et al., 2019). Recent research found a metabolic interaction between ROS and RNS by detecting the peroxyinitrite content and glutathione concentrations in root of wheat (Zhao et al., 2022). Another research found that the plant decreased Cu toxicity through nitric oxide (NO)-mediated pathways to modulate reactive ROS accumulation and oxidative damage (Xu et al., 2022). However, all RNS family members derive from NO. RNS is emerging as a topic of great interest with the increase in the reports about various functions of RNS in plants.

For the initial years after its discovery, NO was extensively studied in animals for its potential neurological functions. NO’s role in plants gradually turned from being a reactive species to its involvement in stress response/signaling, plant growth, and development (Zhou et al., 2021b). It is well-known that several phytohormones are essential components for the regulation of these processes, such as gibberellins (GA), brassinosteroids (BRs), abscisic acid (ABA), auxins, ethylene (ET), jasmonic acid (JA), and salicylic acid (SA). For instance, ABA and GA were reported previously for inducing seed germination. In recent years, NO and sodium nitroprusside (SNP) were reported to regulate the seed germination of apple embryos through phyto-hormonal crosstalk (Xiao-Ping and Xi-Gui, 2010; Krasuska et al., 2017). It is still not clear whether the mechanism of GA- and NO-promoting germination acts antagonistically or synergistically. Abiotic stresses adversely affect the plant growth and development by producing ROS, and regulating phytohormones, signaling, and metabolism in plants. Moreover, NO has been reported to improve the salt tolerance of plants by activating superoxide dismutase (Klein et al., 2018), waterlogging tolerance in wheat (Li et al., 2021), and decrease in toxic ion accumulation (Gadelha et al., 2017). Further research about the concerted participation between NO and phytohormones in signal pathways under environmental stress, would provide data for generating new eco-agricultural technologies based on the regulation of signal molecules for the plants.

**FIGURE 1:**
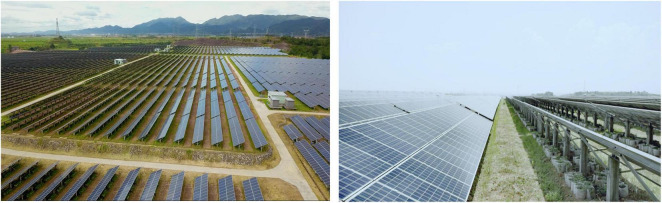
The photovoltaic planting base of Zhejiang Guangsheng Group Co., Ltd. for planting *Tetrastigma hemsleyanum*.

*Tetrastigma hemsleyanum* Diels et Gilg is a perennial herbal plant distributed in subtropical areas of China. Wild *T. hemsleyanum* plants are close to deracinate due to over exploitation and environmental deterioration. Recently, it has become one of the most important varieties of artificial planting medicinal materials in Zhejiang province (Ji et al., 2021). At present, the technology of the greenhouses photovoltaic solar energy construction has been applied in planting *T. hemsleyanum* (Figure 1). Herein, we investigated the impact of photovoltaic condition on endogenous hormone composition and transcriptome profile of *T. hemsleyanum*. The role of NO effectors and/or phytohormones in mediating the photosynthetic adaptability was evaluated in terms of photosynthetic parameters, ROS accumulation, antioxidase activities, and gene expression level of CO_2_ assimilation enzymes. The effect of phytohormones on endogenous NO burst triggered by photovoltaic adaptation was also explored. The results could provide reference for the application of phytohormones/other signaling molecules in photovoltaic agriculture.

## Materials and Methods

### Plant Materials and Growth Conditions

Healthy cutting seedlings of *T. hemsleyanum* were grown under standard greenhouse conditions set as follows: 16 h day/8 h night cycle, 23 ± 2°C temperature, 50% relative humidity, and 250 μmol m^–2^s^–1^ light intensity. Then, 6 months seedlings showing similar growth potential were selected and uniformly divided into two groups, GZ (covered with transparent glass plate) and GF (covered with photovoltaic panel). The photovoltaic panel (ODA-60M) was provided by Ningbo Osda Solar Co., Ltd., China. Each treatment was repeated three times with ten plants. Phenotypic changes were observed and biochemical indexes were determined at 15, 30, and 45 days, respectively. The leaves were sampled on 30 days, frozen in liquid nitrogen immediately, and stored at −80°C for phytohormones and gene expression analysis. The experiment was repeated three times, and each biological replicate consisted of a sample pool from 10 seedlings.

### Exogenous Sodium Nitroprusside and Phytohormones Treatment

Based on our pre-experiment, plants of GF group were either sprayed (foliar application 2 times every 24 h, the application amount was 10 ml for every pot for each time) with ultrapure water or with 100 μM NO-donor SNP or with 300 μM NO scavenger 2-(4-carboxyphenyl)-4,4,5,5-tetramethylimidazoline-1-oxyl-3-oxide (cPTIO) or with phytohormones (50 μM SA, 50 μM MeJA, 50 μM ABA, or 50 mg/L GA, used independently). The treated plants were sampled for detection and analysis after 15 days of treatment. Each test included five seedlings, and three replicates were performed for each treatment.

### Determination of Photosynthetic Efficiency

Five seedlings of the two groups were randomly selected and ten leaves at the same position of plant were sampled. The photosynthetic and fluorescence parameters of *T. hemsleyanum*, such as net photosynthetic rate (Pn), transpiration rate (Tr), stomatal conductance (Cond), mesophyll intercellular CO_2_ (Ci), and the maximum light energy conversion efficiency (Fv/Fm), were determined using the LI-6400 portable photosynthesis meter (Li-COR company, United States) and PAM-2500 portable chlorophyll fluorescence meter (WALZ company, Germany), respectively. Five seedlings of each group were randomly selected, respectively, then values of the upper, middle, and lower leaves were determined uniformly, and the average value was used to reflect the value of this sample. Three replicates were performed for each treatment.

### Determination of Antioxidative Capacity and Fluorescent Imaging of H_2_O_2_, O^2–^, and Reactive Oxygen Species

Approximately 2 g of leaves were homogenized in 3 ml of 50 mM potassium phosphate buffer (pH 7.0). The supernatant was collected by centrifugation at 10,000 × g for 10 min at 4°C, and then be used for the activity determination of peroxidase (POD, EC 1.11.1.7), catalase (CAT, EC 1.11.1.6), superoxide dismutase (SOD, EC 1.15.1.1), and malondialdehyde (MDA) content. POD activity was determined as guaiacol oxidation by H_2_O_2_. SOD activity was analyzed based on the inhibiting rates of the reduction of nitro blue tetrazolium (NBT), CAT activity was determined as the H_2_O_2_ consumption, MDA content was extracted and determined by the thiobarbituric acid reaction method, according to our previous report (Peng et al., 2019).

Leaf and root samples were used to detect the presence of *in situ* accumulation of superoxide and H_2_O_2_ by staining with NBT and 3,3-diaminobenzidine (DAB), respectively. Then, 2.0–2.5 cm of the roots were immediately immersed in a 0.05% solution of NBT or an aqueous solution of 1 mg/ml DAB in 50 mM potassium phosphate buffer (pH 6.4), vacuum infiltrated, and incubated for 12 h in the dark, according to the previous report (Hu et al., 2018; Recalde et al., 2018). Stained segments were washed 3 times by water before photography. Furthermore, 10–15 individuals randomly sampled from each group were used for each experiment.

### Quantification of Phytohormones

Approximately 30 g of each fresh sample was harvested and rapidly frozen in liquid nitrogen, and homogenized into powder. The extraction was conducted by methanol/water/formic acid (15:4:1, V/V/V), 10 μl internal standard mixed solution (100 ng/ml) was added as internal standards, followed by evaporation to dryness, dissolved in 100 μl 80% methanol (V/V), and filtrated through a 0.22 μm membrane filter before liquid chromatography-mass spectrometry (LC-MS/MS) analysis. The extracts were detected using an ultra-high performance liquid chromatography-mass spectrometry (UPLC-ESI-MS/MS) system (UPLC, ExionLC™ AD; MS, Applied Biosystems 6500 Triple Quadrupole). A Waters ACQUITY UPLC HSS T3 C18 (2.1 mm × 100 mm × 1.8 μm) was used. Solvent system were water (0.04 acetic acid): ACN (0.05% acetic acid). The gradient program was as follows: 95:5 V/V at 0–1 min, 5:95 V/V at 1–8 min, 5:95 V/V at 8–9 min, and 95:5 V/V at 9.1–12 min.

The effluent was alternatively connected to acquire linear ion trap (LIT) and triple quadrupole (QQQ) scans on an electrospray ionization (ESI)-triple quadrupole-LIT (Q TRAP)-MS, AB 6500 Q TRAP LC/MS/MS system, and controlled by Analyst 1.6 software (AB Sciex). The ESI source operation parameters were as follows: ion source, ESI ±; source temperature 550°C; ion spray voltage (IS) −4,500 V (negative), 5,500 V (positive); the collision gas (CAD) was medium; and curtain gas was 35.0 psi. A specific set of multiple reaction monitoring (MRM) transitions was monitored to analyze. Phytohormones contents were quantified by Multiquant 3.0.3 software (Sciex) based on the AB Sciex QTRAP 6500 LC-MS/MS platform. Three replications of each sample were performed.

### Measurement of Nitric Oxide and Reactive Oxygen Species Production by Confocal Microscopy

To monitor NO and ROS production in *T. hemsleyanum*, root tips (1 cm) were incubated in darkness for 2 h in the presence of 10 μM fluorescent probe DAF-FM DA (4-amino-5-methy-lamino-2, 7-diamino-fluorescein diacetate, sigma) or 10 μM 2′,7′-Dichlorodihydrofluorescein diacetate (DCFH-DA) prepared in 50 mM Tris–HCl (pH 7.4). It was shaken every 3–5 min during the incubation, and then, washed three times for 10 min with fresh buffer. About 10–15 individuals randomly sampled from each group were used for each experiment. Fluorescence signals were observed using a Leica laser scanning confocal microscopy (LEICA TCS SP8, Germany), and monitored with an excitation line from 495 nm and an emission window from 515 nm. Fluorescence intensity was analyzed using ImageJ software. Fluorescence intensity was quantified by determining green pixels in a defined area. Fluorescence intensity was observed from five micrographs for each treatment.

### RNA Extraction and Transcriptome Analysis

Total RNA was extracted with Plant RNA Reagent (Invitrogen, Carlsbad, CA, United States), and the RNA quality was checked using the kaiaoK5500^®^Spectrophotometer (Kaiao, Beijing, China) and the RNA Nano 6000 Assay Kit of the Bioanalyzer 2100 system (Agilent Technologies, CA, United States). The cDNA was synthesized using a cDNA Synthesis Kit (TaKaRa, Dalian, China). The sequencing of cDNA libraries was performed using the Illumina sequencing system (HiSeq™ 2000, San Diego, CA, United States). Clean reads were obtained using the SOAPnuke (v1.4.0), and were mapped using Bowtie 2(v2.2.3). Fragments per kilo-base of exon per million fragments mapped (FPKMs) were calculated and differential expression analysis was carried out using the DEseq2 (Love et al., 2014). Transcripts with a *p*-adjusted < 0.05 and | log 2-fold change| ≥ 1 were identified as the differentially expressed genes (DEGs). Gene Ontology (GO) and Kyoto Encyclopedia of Genes and Genomes (KEGG) enrichment analyses were performed to reveal the biological functions and the signal transduction pathways of the DEGs, *q* < 0.05 was considered to be significantly enriched.

### Quantitative Real-Time PCR Analysis

Total RNA was isolated from 100 mg (fresh-weight) of leaves using the plant RNA extraction kit (Nanjing Vazyme Biotech Co., Ltd.) and synthesis of cDNA was performed with Evo M-MLV Mix Kit with gDNA Clean for qPCR AG11728 (Accurate biotechnology (Hunan) Co., Ltd.). Each reaction contained 10 μl of 2xSYBR Green Pro Taq HS Premix AG11701 (Accurate biotechnology (Hunan) Co., Ltd.), 2 μl of template cDNA, 0.4 μl of forward and reverse primers each (10 μM). GAPDH gene was used as an internal reference (Table 1). The PCR reaction procedure was as follows, incubation at 95°C for 2 min followed by 40 cycles of 95°C for 5 s and 60°C for 30 s. Each gene was tested in three biological replicates, with three technical repeats. The expression level for each sample was expressed as 2^–ΔΔCt^. The data were exhibited as the mean ± SD of three independent experiments.

**TABLE 1:** Primers sequences used for quantitative real-time PCR analysis (qRT-PCR) for detecting gene expression.

Gene annotation	Primer F (5′–3′)	Primer R (5′–3′)	Tm	Length (bp)
Photosystem I reaction center subunit XI (psaL)	CTTCACTTCACCATCAACC	GTCTCAAGGCTTCCGATA	60	183
Magnesium-chelatase subunit (ChlI)	CATCCTGCTCGGTTTATTC	CTCTCCTCCACAATCTTCA	60	143
Ferredoxin (petF)	GGAGAAGGAGTTTGAATGC	AAGTCAGAACCCATCCAG	60	194
Photosystem II oxygen-evolving enhancer protein (psbQ)	CCTTGATCTGCCATTGAAG	ACAGTGTTGAGGTCGTAG	60	189
Photosystem I subunit IV (psaE)	ACCTCCATGCTCATCTTC	TCCTTCCTCAGAATCTTCAC	60	194
Carbonic anhydrase (cynT)	GCCTTTCCCAGAACAATG	TCCACCCTTCAATGATAGAG	60	124
Nitrate reductase (NAD(P)H), (NR1)	CGGGAAGGTGTATGATGTA	GAGGAACGCTTGATGAATC	60	179
Nitrate reductase (NAD(P)H), (NR2)	TTCAAGACGGTGGTAGTG	CAAGGAGAGGAACGAAGA	60	144
Abscisic-aldehyde oxidase (AAO)	CAGTTCCAGACGAAGACA	CAGTAGCAACAGGCATTG	60	177
9-cis-epoxycarotenoid dioxygenase (NCED1)	CCACTTGTGAGGTGATAGA	CGTAACGGCTACAGAAGA	60	189
9-cis-epoxycarotenoid dioxygenase (NCED2)	GGAAGGAGACATTGAGACT	GAATCGGAAGTAGGTTAGGA	60	151
Phenylalanine–tRNA ligase beta subunit (pheT)	GATGTGGTTGAGGATGTTG	CAGTGGCTTCAATGATACAG	60	78
Phenylalanine ammonia-lyase (PAL)	GGATGAAGTGAAGCGTATG	GCTGTCTGTACCATTGTTC	60	199
Hydroxymethylbilane synthase (hemC)	TCACGAGGTTACCAGATTAG	GAAGCATATCCAGCAATAGG	60	93
Magnesium chelatase subunit H (CHLH)	CTATGTGGCTGTGATTATGG	CGAACGGCTTCTTAGTAAC	60	128
GAPDH	AGCAGCCTTGTCCTTGTCAGTG	GATTGGACGTTTGGTTGCGAG	60	150

*Tm, melting temperature.*

## Results

### Photovoltaic Condition Decreased Phytohormones Levels, Especially Salicylic Acid and Jasmonic Acid

We comprehensively compared the accumulation of multiple phytohormones between GZ and GF groups to explore the contributions of phytohormones in response to the photovoltaic planting pattern.

**FIGURE 2:**
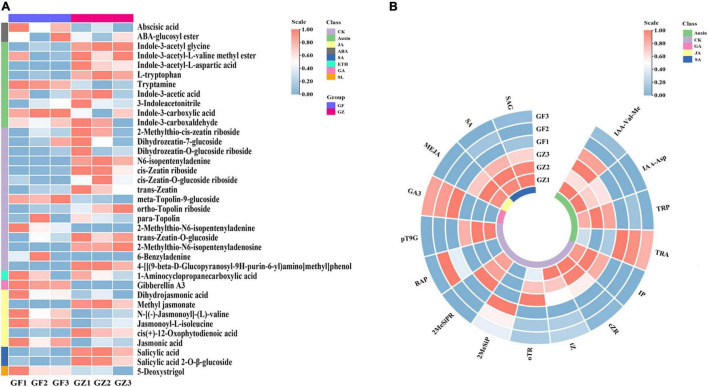
**(A)** The overall heatmap of 8 categories of phytohormones in *T. hemsleyanum*. **(B)** The heatmap of significantly different phytohormones between GZ and GF in *T. hemsleyanum*. The color bar represents the normalized fold change values.

We detected 37 phytohormones grouped into 8 categories (Supplementary Table 1), such as 2 ABA, 9 Auxin, 15 CK, 1 ETH, 1 GA, 6 JA, 2 SA, and 1Soligolactone (SL) (Figure 2A). The differentially accumulated phytohormones (DAP) between GZ and GF were screened using | log 2-fold change| ≥ 1 and *p* < 0.05. A total of 16 DAP were identified in GZ _vs._ GF, such as 4 auxin, 8 CK, 1 GA, 1 JA, and 2 SA. Overall, the levels of ABA, ETH, and SL have no significant differences between GZ and GF. The content of tryptamine increased in the GF, while the levels of other auxins, i.e., indole-3-acetyl-L-valine methyl ester (IAA-Val-Me), indole-3-acetyl-L-aspartic acid (IAA-Asp), and L-tryptophan (TRP) decreased in response to the photovoltaic treatment. The content of JA and SA (such as, SA and SA 2-O-β-glucoside) were significantly decreased by photovoltaic treatment. GF group had higher contents of cytokinins than GZ except for 6-benzyladenine (BAP) and 2-methylthio-N6- isopentenyladenine (2MeSiP). The levels of N6-isopentenyladenine (IP), cis-Zeatin riboside (cZR), trans-Zeatin (tZ), and ortho-Topolin riboside (oTR), which are naturally occurring cytokinin, decreased after photovoltaic treatment. Although the level of GA was significantly induced by photovoltaic treatment, it could hardly be detectable both in GZ and in GF. Overall, we noticed that GF decreased significantly the contents of phytohormones especially in SA and JA, while no significant differences were detected in the case of ABA, ETH, and SL (Figure 2B).

### Transcriptome Analysis Revealed Key Factors Involved in Photovoltaic Adaptation

**FIGURE 3:**
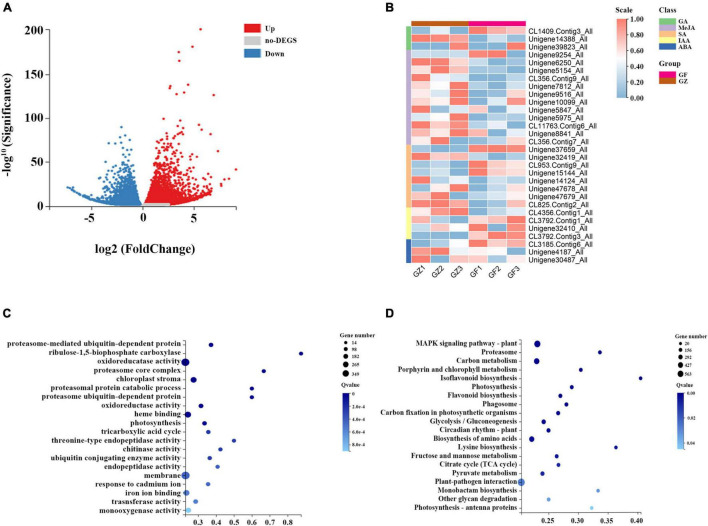
**(A)** Volcano plot of differential gene expression analysis. **(B)** Heatmap of hormone-related genes expression level. **(C)** Gene Ontology (GO) level terms classification of differential genes. **(D)** Kyoto Encyclopedia of Genes and Genomes (KEGG) pathway analysis for differential gene expression analysis between GZ and GF in *T. hemsleyanum*.

A total of 42.43–44.67 million clean reads were obtained, and the Q30 of the raw reads in the 6 RNA-seq libraries ranged from 91.7 to 95.241%. A total of 111,428 unigenes were generated, and the mean length was 1,313 bp with N50 length of 2,167 bp. Based on *de novo* assembly, we found that 7,024 upregulated and 5,591 downregulated genes in GZ _vs._ GF comparison (Figure 3A).

A total of 31 genes related to hormone synthesis were detected, most of them were downregulated in GF group compared with GZ group (Figure 3B). The results of GO functional and enrichment analyses demonstrated that the top-ten enriched GO terms were related to “proteasome-mediated ubiquitin-dependent protein catabolic process,”“ribulose-1,5-bisphosphate carboxylase/oxygenase activator activity,” “oxidoreductase activity,” “chloroplast strom,” “oxidoreductase activity, acting on paired donors, with incorporation or reduction of molecular oxygen, NAD(P)H as one donor,” “heme binding,” “photosynthesis,” etc. (Figure 3C). Furthermore, the DEGs were significantly enriched using the KEGG database. The top enriched KEGG terms contributed by these DEGs included “mitogen-activated protein kinase (MAPK) signaling pathway-plant,” “carbon metabolism,” “porphyrin and chlorophyll metabolism,” “photosynthesis,” bioflavonoid biosynthesis,” “carbon fixation in photosynthetic organisms,” “circadian rhythm-plant,” “photosynthesis- antenna proteins,” etc. (Figure 3D).

The above analyses indicated that the identified DEGs were the most relevant to photosynthesis, MAPK signaling pathway, carbohydrate metabolism, and oxidation-reduction reaction. A total of 441 DEGs were annotated in MAPK signaling pathway, which was the top enriched KEGG term. MAPK cascade plays a prominent role in plant growth, stress response, and signal transduction of hormones. ROS are likely to directly or indirectly activate MAPKs. MAPK activation is an important step in the biosynthesis of various phytohormones. The largest number of DEGs was identified to link to the oxidoreductase activity in the GO enrichment analysis. A transcriptome analysis detected 277 genes encoding multiple enzymes in plant hormone signal transduction pathway. Of these genes, the Auxin signal transduction protein family was the most represented with 42 members, such as 31 Auxin-responsive proteins, 1 Auxin transporter-like protein, and 10 Auxin-induced proteins (Supplementary Table 2).

**FIGURE 4:**
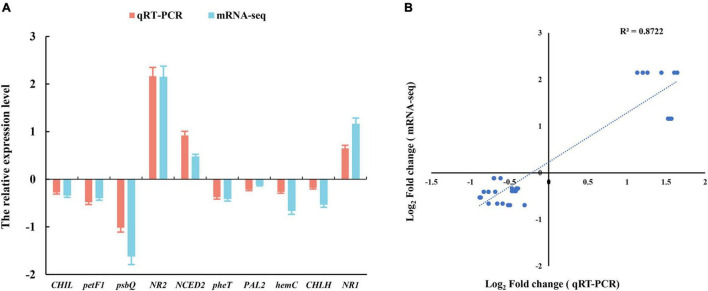
**(A)** Verification of RNA-Seq data by the quantitative real-time PCR (qRT-PCR) assay. **(B)** Pearson’s correlation coefficients of the log2 of gene expression ratios obtained from RNA-seq data and qRT-PCR. All the genes information is showed in Table 1. Three biological replicates and three technical replicates were collected for each sample.

We selected 10 genes from photosynthesis and hormone signaling pathway to assess the relative expression levels by the quantitative real-time PCR (qRT-PCR) analysis. This independent evaluation revealed that the patterns of RNA-Seq expressions on these genes were highly consistent with the qRT-PCR data, and a correlation coefficient (R2) of 90.89% between the two techniques was obtained (Figure 4).

### Exogenous Phytohormones and Nitric Oxide Enhance Photosynthetic Efficiency and Modulate-Related Gene Expression Under Photovoltaic Condition

**FIGURE 5:**
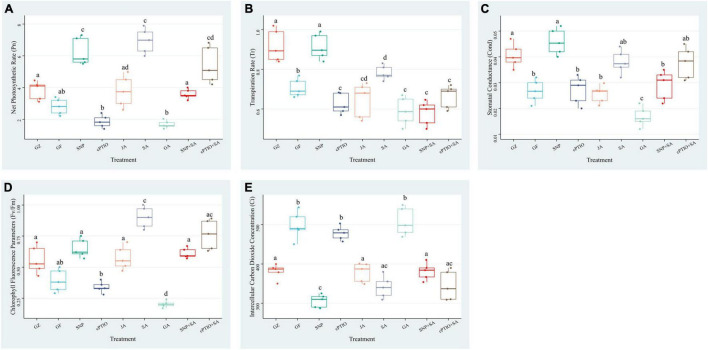
Effect of nitric oxide (NO) effectors and/or phytohormones on **(A)** net photosynthetic rate (Pn), **(B)** transpiration rate (Tr), **(C)** stomatal conductance (Cond), **(D)** chlorophyll fluorescence parameters (Fv/Fm), and **(E)** intercellular carbon dioxide concentration (Ci) of *T. hemsleyanum* under photovoltaic condition (GF) and normal light condition (GZ). Data are means ± DE of the three technical replicates from five seedlings for each treatment. Means that do not share a same letter are significantly different at *p* ≤ 0.05 level according to Tukey’s test.

Exposure to photovoltaic condition reduced the Pn, Tr, Cond, and Fv/Fm by 29.1, 18.0, 26.1, and 27.8%, respectively, compared with GZ group, whereas intercellular carbon dioxide concentration (Ci) showed a significant increase of 26.6%. Although the Pn was significantly lower in GF group than in GZ group, JA supplementation narrowed that gap, SNP and SA treatment even greatly enhanced Pn to 1.52- and 1.76-fold of that of GZ group, respectively. Applying with cPTIO decreased Pn by 33.2% compared with GZ group. The enhancement effect of the combination of NO quencher cPTIO and SA treatment was decreased by 22.9% than that of SA treatment alone. However, when plants were treated with the combination of “SA + SNP,” the enhancement effect of SA on Pn was markedly counteracted by 49.4%. On the contrary, GA showed no significant effect on the Pn level. Similar trends were noted for Tr, Cond, and Fv/Fm. On the other hand, Ci was increased under photovoltaic condition when compared with control. Exogenous SA, SNP, and JA alleviated the increment, and the combined treatment of SA + SNP showed a higher Ci level than the combined treatment of SA + CPTIO (Figure 5). These data indicated that NO, JA, and SA treatment alone significantly enhanced the photosynthetic efficiency which was decreased by exposure to photovoltaic condition, but the combined treatment of NO + SA could weaken the enhancement effect.

### Responses of Photosynthesis-Related Genes

**FIGURE 6:**
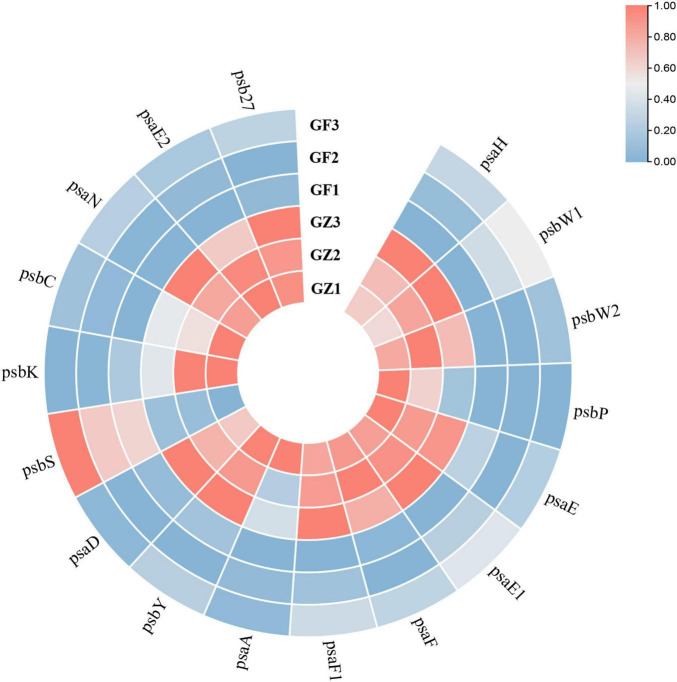
The heatmap of significantly different genes encoding proteins involved in photosystem I (PS I) and photosystem II (PS II) reaction center between GZ and GF in *T. hemsleyanum*. The color bar represents the normalized fold change values.

Based on the transcriptome information, we investigated the whole genome expression level of the genes involved in photosynthesis. A total of 79 unigenes encoding multiple proteins or enzymes in the chlorophyll and photosystem biosynthesis and metabolism pathway (Supplementary Table 3). From Figure 6, we could see that a majority of genes involved in photosynthesis (69/79) were specifically downregulated in the GF group, compared with the GZ group. A total of 17 key genes encoding proteins involved in photosystem I (PS I) and photosystem II (PS II) reaction center (Supplementary Table 4), among them 16 genes were downregulated, such as PS I reaction center subunit IV B (Unigene48386), PS I reaction center subunit VI (CL5676.Contig2), and PS II oxygen-evolving enhancer protein (CL2591.Contig1) of GF were repressed to 31.3, 53.4, and 42.3% of GZ, respectively (Figure 6). In particular, 3 of 8 unigenes encoding psbP domain-containing protein were almost completely not expressed. The above results indicated that photosynthetic electron transport machinery was repressed to a certain degree in response to GF treatment.

**FIGURE 7:**
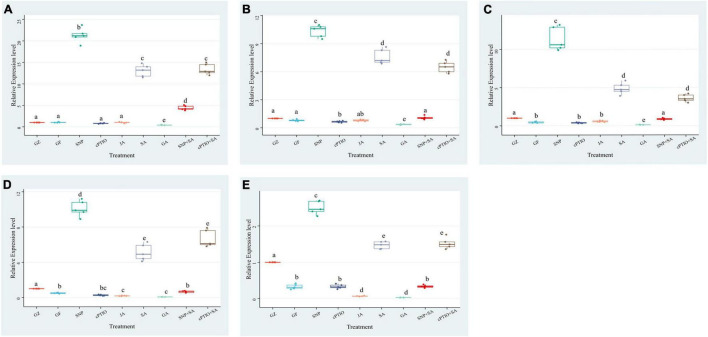
Effect of NO effectors and/or phytohormones on expression levels of **(A)** *psaL*, **(B)** *CHIL*, **(C)** *petF1*, **(D)** *psbQ*, and **(E)** *psaE*, of *T. hemsleyanum* under photovoltaic condition (GF) and normal light condition (GZ). Data are means ± SE of the three technical replicates from five seedlings for each treatment. Means that do not share a same letter are significantly different at *p* ≤ 0.05 level through Tukey’s test.

To confirm the effect of photovoltaic condition and each treatment on photosynthetic efficiency, an RT-PCR was performed to determine the relative expression levels of 5 key genes in the photosystem biosynthesis and metabolism pathway: *psaL, CHIL, petF1, psbQ*, and *psaE* (Figure 7). Although 5 key genes showed different degrees of expression changes among each group, they generally have a similar variation trend. The expression levels of the GF group were below or close to the GZ group. SNP and SA treatment dramatically increased genes expression levels, while the GA and cPTIO treatment significantly lower their expression levels. The difference relative transcript level of *psaL* reached a maximum at 21.3 in “SNP vs. CK” and 13.5 in “SA vs. CK,” respectively, while the difference multiple of “GA vs. CK” and “JA vs. CK” was 0.40 and 1.01, respectively. It suggested that *psaL* was inhibited by GA but were not significantly influenced by JA. Surprisingly, SA + SNP combined treatment only upregulated the gene expression level by 4.4-fold compared with CK, it was obviously lower than that in the SNP or in SA individual treatment group. On the other hand, cPTIO + SA combined treatment up-regulated the gene expression level by 13.3-fold, close to the SA treatment group. It suggested that SA had no synergistic effect with NO in promoting the expression level of *psaL*, instead, an antagonistic link between them might even be possible. The expression of another gene, *psbQ*, also showed 9.9- and 5.1-fold upregulation under SNP and SA treatment, respectively. The difference multiple of “SNP + SA vs. CK” and “cPTIO + SA vs. CK” reached 0.65 and 6.6, respectively. It suggested that the inhibition of NO generation is beneficial for the activating effect of SA on *psbQ*.

### Exogenous Phytohormones and Nitric Oxide Treatment Mitigated the Accumulation of Reactive Oxygen Species and Potentiated Antioxidant Capacity

**FIGURE 8:**
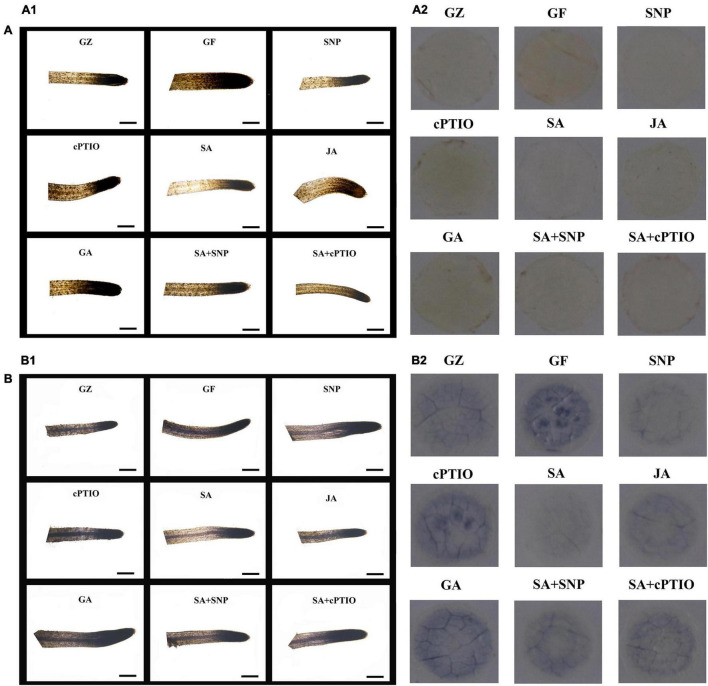
*In situ* accumulation of H_2_O_2_ and O^2–^ by 3,3-diaminobenzidine (DAB) **(A)** and nitro blue tetrazolium (NBT) **(B)**. Staining of the roots **(A1,B1)** and leaves **(A2,B2)** after the respective treatment of NO effectors and/or phytohormones under the photovoltaic condition (GF) and the normal light condition (GZ). The figure is representative of ten segments stained in each experiment.

The NBT- and DAB-staining were performed to show H_2_O_2_ and O^2–^ accumulation, respectively. Results of Figure 8 revealed more intense brown or dark blue patches on the roots/leaves of the GF group than on those from the GZ group, and indicated overproduction of H_2_O_2_ and O^2–^ in response to the photovoltaic condition. A similar amount of H_2_O_2_ and O^2–^ was observed in GA-treatment seedlings, compared with the GF group. However, SA, SNP, or JA treatment alone considerably diminished the H_2_O_2_ and O^2–^ accumulation in the leaves/roots of the GF seedlings. This indicated that the SA-, JA-, and NO-mediated response was closely coupled with the H_2_O_2_ and O^2–^ level. On the other hand, applying cPTIO to quench endogenous NO would increase the accumulation of H_2_O_2_ and O^2–^ in seedlings. Similarly, accumulation of H_2_O_2_ and O^2–^ was promoted by cPTIO + SA combined treatment as compared with the SA treatment alone. But the leaves/roots of SNP + SA combined treatment group were stained more deeply by the two chemicals than those in SA or SNP treatment alone group, indicating more amount of H_2_O_2_ and O^2–^ was produced when SA coexisted with NO. These data indicated that NO, JA, and SA alleviated H_2_O_2_ and O^2–^ production, while GA and NO quencher led to the overproduction of H_2_O_2_ and O^2–^, NO donor could weaken the scavenging effect of SA.

**FIGURE 9:**
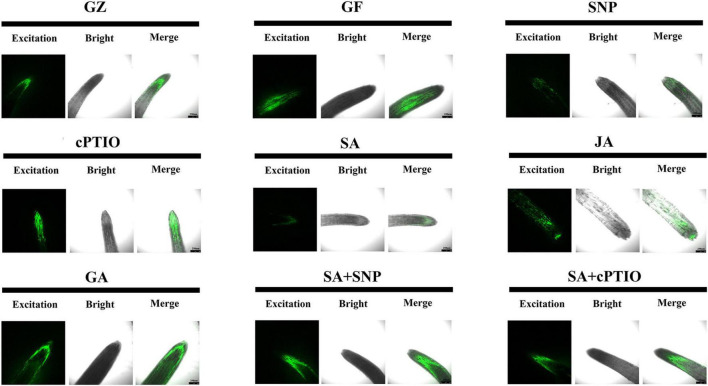
Reactive oxygen species (ROS) accumulation of the roots after the respective treatment of NO effectors and/or phytohormones under the photovoltaic condition (GF) and the normal light condition (GZ), using 10 μM DCFH-DA as a fluorescent probe. The figure is representative of ten segments stained in each experiment.

The DCFH-DA staining was performed to show ROS accumulation, respectively. Results of Figure 9 revealed more intense green fluorescence on the roots of GF group than on those from the GZ group, and indicated overproduction of ROS in response to photovoltaic condition. However, SA, SNP, or JA treatment alone considerably diminished an ROS accumulation in the roots of the GF seedlings, especially SA and SNP. This indicated that the SA-, JA-, and NO-mediated response was closely coupled with the ROS level. Similarly, accumulation of ROS was promoted by cPTIO + SA combined treatment as compared with the SA treatment alone. However, the roots of SNP + SA combined treatment group were stained more deeply by the two chemicals than those in SA or SNP treatment alone group, indicating that more amount of ROS was produced when SA coexisted with NO. These data indicated that NO, JA, and SA alleviated ROS production, while GA and NO quencher led to the overproduction of ROS. NO donor could weaken the scavenging effect of SA.

**FIGURE 10:**
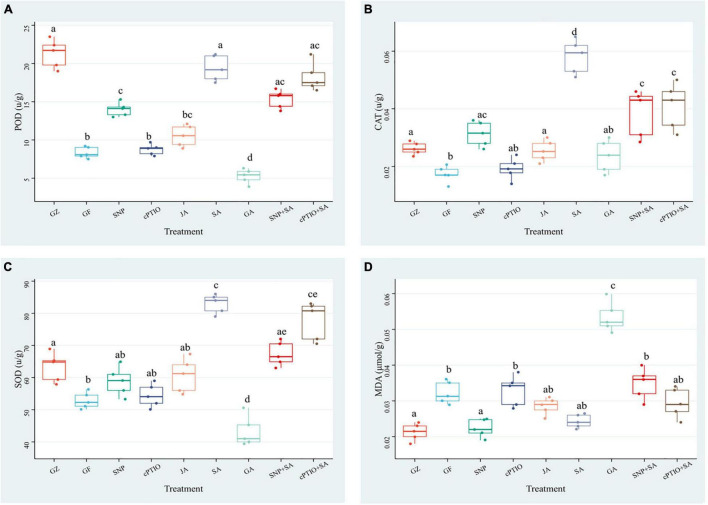
Effect of NO effectors and/or phytohormones on the activities of determination of peroxidase (POD) **(A)**, catalase (CAT) **(B)**, superoxide dismutase (SOD) **(C)**, and the content of malondialdehyde (MDA) **(D)** in *T. hemsleyanum* under the photovoltaic condition (GF) and the normal light condition (GZ). Data are means ± SE of the three technical replicates from five seedlings for each treatment. Means that do not share a same letter are significantly different at *p* ≤ 0.05 level through a Tukey’s test.

The aforementioned results suggested that ROS accumulation varied among the photovoltaic condition and different treatments. Since enzymatic scavenging had an important influence in ROS accumulation, investigation of the antioxidant system was thus conducted by measuring the activities of SOD, CAT, and POD. The three enzymes exhibited almost the same trends among different treatment groups, although they differed in the order of magnitude. Under the photovoltaic condition, a considerable (*p* ≤ 0.05) decrease by 60.8, 33.9, and 16.4% was observed in the activities of SOD, CAT, and POD enzymes, and a considerable (*p* ≤ 0.05) increase by 47.0% was observed in the content of MDA yet, compared with normal illumination group (Figure 10). Exogenous application of SNP, SA, and JA significantly (*p* ≤ 0.05) improved the POD activity by 67.8, 100.4, and 26.3%, respectively, they also improved the CAT activity by 80.8, 215.3, and 46.8%, respectively. While the SOD activity was improved by 56.0% only in response to SA. Application of cPTIO alone had no perceptible effect on the antioxidant system, but applying with cPTIO and SA combination counteracted the enhancement effect of SA on the activity of POD, SOD, and CAT by 5.9 (*p* > 0.05), 5.8% (*p* > 0.05), and 29.6% (*p* ≤ 0.05), respectively. The SNP + SA combined treatment further counteracted the enhancement effect of SA on the activities of POD, SOD, and CAT by 20.8% (*p* ≤ 0.05), 18.3% (*p* > 0.05), and 33.6% (*p* ≤ 0.05), respectively. On the other hand, GA-treated samples had the lowest antioxidant enzyme activity and the highest MDA content among all the treatments. The above results had a good agreement with the ROS accumulation dynamics (Figure 9).

The results of the antioxidant system demonstrated that the photovoltaic condition significantly increased ROS accumulation and decreased the antioxidant capacity. The antioxidant enzyme activities were consistent with the NO, SA, and JA levels, but negatively correlated with the ROS accumulation, thereby suggesting that NO, SA, and JA had protective roles, but the combined treatment of NO + SA could weaken the protective effect.

### Phytohormones Reduced Endogenous Nitric Oxide Burst Triggered by Photovoltaic Adaptation

**FIGURE 11:**
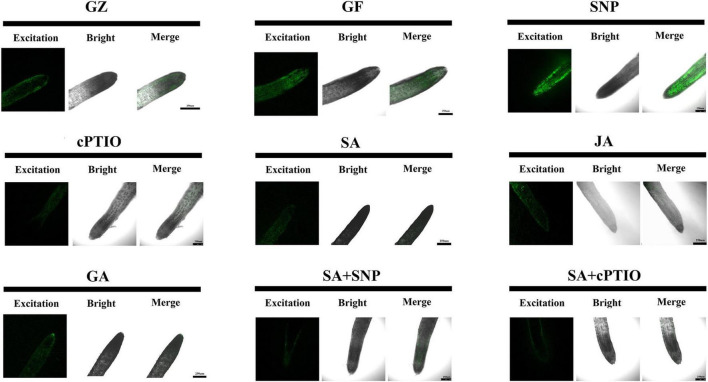
Nitric oxide (NO) accumulation in roots of *T. hemsleyanum* after the respective treatment of NO effectors and/or phytohormones under the photovoltaic condition (GF) and the normal light condition (GZ), using 10 μM DAF-2DA as a fluorescent probe. The figure is representative of ten segments stained in each experiment.

Semi-quantitative measurement of NO generation in root tips after different treatments was performed using the fluorescent probe 4,5-diaminofluorescein diacetate (DAF-2DA). As shown in Figure 11, the level of NO was 11.2% higher under the photovoltaic condition than under the normal light condition. This suggests that the production of endogenous NO was triggered by photovoltaic adaptation. Moreover, SNP strongly increased the level of NO, reaching an approximately 22.0 and 35.4% higher level in relation to the normal light and photovoltaic condition groups, respectively. In contrast, the photovoltaic condition-induced endogenous NO burst was weakened by treatment with the NO scavenger cPTIO, the level of NO decreased by 26.0%. The results suggested that NO was involved in photovoltaic condition-induced ROS accumulation and boosted photosynthesis in *T. hemsleyanum*.

**FIGURE 12:**
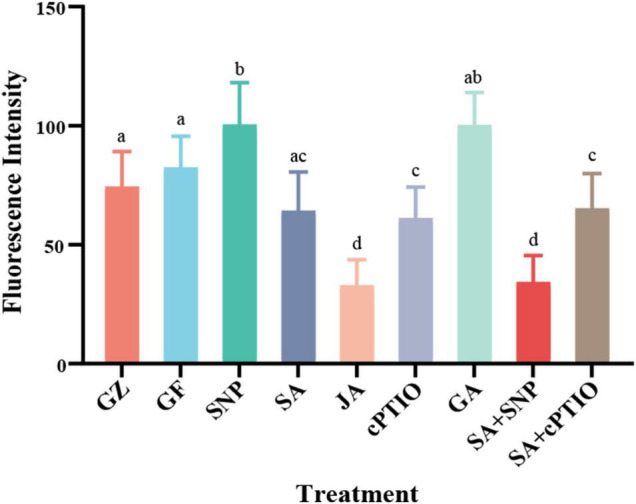
The values of fluorescence intensity. Means in that do not share a same letter are significantly different at *p* ≤ 0.05 level through Tukey’s test.

To gain further insight into the possible interaction between NO generation and phytohormone signaling in response to the photovoltaic condition, the *in situ* NO level regulated by several kinds of phytohormones was analyzed. All phytohormones, except GA, significantly decreased the storage of NO message, especially under the SA treatment. The level of NO decreased by 22.3% under the SA treatment, similar to that of cPTIO treatment, while SNP increased the level of NO by 22.0%, however, SNP + SA combined treatment significantly strengthened the inhibitory effect on NO accumulation by 58.7% (*p* ≤ 0.05), similar to that of JA treatment (Figure 12).

## Discussion

Greenhouse horticulture is essential in satisfying the increasing global food demand under climatic variation situation. As an increasingly important clean source of energy, solar photovoltaic energy system has attracted much attention. Due to the large land occupation of photovoltaic panel, it is economic to develop the photovoltaic planting pattern under photovoltaic panels. Research about energy-efficient cover materials on yield and photosynthesis have been examined in main vegetables, such as tomato (Ezzaeri et al., 2018) and cucumber (Alsadon et al., 2016). The result indicated that the covers had significant effects on photosynthate allocation, yield, and the nutrient composition in vegetables. However, the potential molecular and physiological mechanisms of photovoltaic response in horticultural crops are rarely studied.

In this study, exposure to the photovoltaic condition reduced the photosynthetic efficiency, decreased phytohormones levels, provoked endogenous NO burst, and disrupted the redox homeostasis of *T. hemsleyanum* and the transcriptome analysis verified that the largest number of DEGs were the most relevant to photosynthesis, plant hormone signal transduction pathway, MAPK signaling pathway, and oxidation-reduction reaction.

Mitogen-activated protein kinase cascade plays an important role in plant growth, stress response, and signal transduction of hormones. ROS are likely to directly or indirectly activate MAPKs. There are increasing experimental evidence that various gaseous signaling molecules are involved in MAPK cascades. NO could activate MAPK cascades during stress, and MAPK cascades could also mediate NO-dependent physiological processes (Wu et al., 2021).

It has been proved that various environmental stresses might cause many physiological disorders of plants, such as a decline in biomass production, chlorophyll content, and photosynthetic rate, directly or indirectly, through oxidative damage. A frequent occurrence is the generation of reactive chemical species, such as RNS and ROS. Numerous studies have been conducted to explore the roles of ROS as signaling molecules or as oxidative burst causing-agents. The RNS biology, however, is emerging as recent research hotspot. NO was thought to be an important precursor of RNS members. In this study, the photovoltaic condition provoked endogenous NO and ROS burst, similar to the responses to environmental stress. However, photovoltaic condition-induced ROS accumulation could be alleviated by exogenous NO to the upregulating activities of CAT, POD, and SOD, thus resulting in the alleviation of oxidative damage, while the NO inhibitor cPTIO reversed these responses. These responses are in agreement with several previous findings. Similar protective effects were obtained for *Pistia stratiotes* under arsenic stress (Farnese et al., 2017). NO donor SNP pretreatment conferred salt and drought tolerance by the activation of antioxidase (SOD, POD, and CAT) and lowering the oxidative stress, while the inhibitor L-NAME or cPTIO application made the seedlings more sensitive to both the two types of stresses (Fan and Liu, 2012; Kaya and Ashraf, 2021).

Sodium nitroprusside significantly increased the photochemical efficiency, alleviated the decrease of the Chl content, and decreased NPQ of PSII of ryegrass seedling leaves under alkaline stress (Procházková et al., 2013). NO has been reported to regulate the chlorophyll production (Romero-Puertas et al., 2004). SNP treatment decelerated chlorophyll loss and improved the photosynthetic efficiency under either dehydration or drought stress (Fan and Liu, 2012). NO plays an important role in CO_2_ elevation-induced stomatal closure, and therefore enhance the photosynthesis of Arabidopsis, while NO inhibitor tungstate and L-NAME significantly inhibited stomatal closure by scavenging NO and reversed the enhancement of photosynthesis (Wang et al., 2015). In our study, the photovoltaic condition significantly decreased Fv/Fm, Pn, Tr, and Cond, and increased Ci. The presence of GA or cPTIO intensified the degree of change, while SNP, SA, and JA alleviated the decline of photosynthesis in different degrees, especially SNP and SA. Transcriptome analysis revealed that a majority of genes (69 of 79) involved in was specifically down-regulated in response of the photovoltaic condition, such as *psaL, CHIL, petF1, psbQ*, and *psaE*, and these key genes were proved to be upregulated by the SNP-treatment and downregulated by the cPTIO-treatment. The results suggested that both NO and SA play a significant role in recovering the suppression of photosynthesis induced by photovoltaic condition. It is surprising that the integration of NO and SA treatment provides no additional functionality, but it significantly weakened those restoration effects, much lower than NO or SNP treatment alone, than cPTIO and SA treatment. It indicates that there is some kind of antagonism between NO and SA. These conclusions are further confirmed by expression levels determination of the key genes for photosynthesis. The key gene expression levels of SNP + SA combined treatment were obviously lower than that in SNP or in SA individual treatment group. On the other hand, cPTIO + SA combined treatment upregulated the gene expression level, and close to the SA treatment group. It suggested that the inhibition of NO generation is beneficial for the activating effect of SA on photosynthesis.

Nowadays, the application of phytohormones/growth regulators has become an effective approach to regulate the plant metabolism and strengthen plant tolerance against various stresses. Not only synergistic but also antagonistic interactions between phytohormones and other signaling molecules have been reported to mediate the tolerance mechanisms of plants. Over the last decade, intricate crosstalk between NO and phytohormones during the regulation of several plant responses have been described by some studies. NO increased lettuce’s resistance to salt stress by regulating its hormonal balance. NO and CKs showed positive interaction in regulating photosynthesis and improving adaptability of *Zea mays* to drought stress (Mishina et al., 2007). *In vivo* NO detection showed that NO generation inside the guard cells is important for ABA-regulated stomatal closure (Neill et al., 2002). Both antagonistic and positive promotion effect between NO and GAs has been observed for several. NO could inhibit the GA synthesis by reducing the expression levels of GA20 oxidase3 (GA20ox3), which encodes a key enzyme in the synthesis pathway of active GAs. Moreover, NO promotes the expression of DELLA proteins, which are repressors of GA signaling (Lozano-Juste and León, 2011). On the contrary, under low- and high-phosphorus levels, NO showed antagonistic action against GA in regulating the primary root growth of Arabidopsis thaliana (Wu et al., 2014). Exogenous NO decreased the detrimental influence of overproduced ET under abiotic stresses by inhibiting 1-aminocyclopropane-1-carboxylic acid (ACC) synthase and ACC oxidase activities, which were responsible for the production of ET (Manjunatha et al., 2010). Under salt stress, the synergistic interaction between SA and NO was found in decreasing the damages in Soybean plants by more significantly activating CAT, ascorbate peroxidase (APX), and guaiacol peroxidase (GPX), and the protective action of SA + SNP combined treatment was more effective than that of SA and SNP alone (Simaei et al., 2011). On the contrary, our study demonstrated that the photovoltaic condition significantly increased ROS accumulation and decreased antioxidant capacity. NO, JA, and SA alleviated ROS production, while GA and NO quencher led to overproduction of ROS. The protective action of SA + SNP combined treatment against oxidative damage induced by photovoltaic condition was often less efficient than effects of SA and SNP alone. Similarly, SA was reported to antagonize the ability of NO to decline respiration, thereby causing oxidative stress (Fatima et al., 2021).

The above results indicated that although the crosstalk mode among NO and other signaling compounds was not always synergistic, sometimes antagonist responses under unfavorable situations. The NO crosstalk response under similar stress would vary plant by plant due to the complex signaling cascades of signaling compounds and their interacting signals. Therefore, future research considering the interaction between multiple phytohormones and NO under environmental stress is required to elucidate the biochemical mechanisms of how hormones interact with NO to fine tune the plant metabolism.

So far, little is known about the upstream and downstream relation between NO and phytohormones in the environmental response as indicated by a series of studies in this field with often conflicting results. NO and SA synergistically interact to ameliorate oxidative damage and alleviate salt/osmotic stress, and NO signaling action may occur downstream of SA (Liu et al., 2014; Naser et al., 2014). Increased Cd tolerance of Arabidopsis thaliana was mediated by auxin-induced NO accumulation (Xu et al., 2011).

The composition and content of hormones were altered in response to high saline conditions and they trigger NO production. Thus, after NO production, the balance of phytohormones will be rebuilt through feedback mechanisms (Nawaz et al., 2017). Under heat stress, exogenous melatonin increased the NO burst along with the expression of nitrate reductase- and NO synthase-related genes, which facilitated the scavenging of excess ROS and confronting oxidative damage induced by the heat stress in tomato seedlings. On the contrary, NO has been considered to be one of the first molecules in response to Cd stress, which in turn suppressed AUX carriers and AUX accumulation, resulting in inhibition of root elongation in Arabidopsis seedlings (Yuan and Huang, 2016). Some studies have shown that NO levels were unchanged or even low in mutant/transgenic plants with the higher level of CK. However, other reports have revealed CK treatment increased NO production (Freschi, 2013). CKs reduced NO generation in guard cells and stimulated stomatal opening. Moreover, CKs even abolished NO generation and thereby facilitated reopening of closed stomata under dark conditions. SA prevented NO accumulation in Arabidopsis under salt stress conditions indicating an antagonistic relationship between NO and SA (Khokon et al., 2011). In our study, all phytohormones, except GA, significantly decreased an endogenous NO burst triggered by photovoltaic adaptation, especially under an SA treatment. SA might also be a potent scavenger of NO and counter the restoration effect of NO on growth and photosynthetic potential in *T. hemsleyanum*.

To sum up, maintaining hormone homeostasis is a pressing challenge for plants under stress conditions, the NO crosstalk with various phytohormones in plant growth regulation in response to stress conditions is ever-present and very complicated, and our knowledge of crosstalk signaling pathways is fragmentary so far, most known signaling have not been connected to stress sensors. NO influences on the transcriptional regulation of gene encoding hormone-associated proteins are far from being clarified. Further, the large number of potential targets of NO action would be an important challenge for future research on the signaling networks of NO–phytohormone crosstalk in plant responses to environment, which would reveal new ideas to manipulate the responses of plant to adverse environmental conditions under the background of global climate change.

## Data Availability Statement

The datasets presented in this study can be found in online repositories. The names of the repository/repositories and accession number(s) can be found in the article/Supplementary Material.

## Author Contributions

XP and LG conceived the study. ML performed GO and KEGG pathways enrichment analysis and designed the experiments. ZX performed physiological indicators determination and endogenous hormone determination. CY performed RNA extraction and quality determination. ZZ and YW carried out the analysis. All authors have read and approved the manuscript.

## Conflict of Interest

The authors declare that the research was conducted in the absence of any commercial or financial relationships that could be construed as a potential conflict of interest.

## Publisher’s Note

All claims expressed in this article are solely those of the authors and do not necessarily represent those of their affiliated organizations, or those of the publisher, the editors and the reviewers. Any product that may be evaluated in this article, or claim that may be made by its manufacturer, is not guaranteed or endorsed by the publisher.

## Supplementary Material

Supplementary Table 1 All phytohormones identified in *Tetrastigma. hemsleyanum* in GZ and GF groups.

Supplementary Table 2 All differentially accumulated genes encoding multiple enzymes in plant hormone signal transduction pathway between GZ vs. GF group.

Supplementary Table 3 All differentially expressed genes (DEGs) encoding multiple well-known proteins or enzymes in the chlorophyll and photosystem biosynthesis and metabolism pathway between GZ vs. GF group.

Supplementary Table 4 All DEGs encoding proteins involved in photosystem I (PS I) and photosystem II (PS II) reaction center between GZ vs. GF group.
